# VMCast: A VM-Assisted Stability Enhancing Solution for Tree-Based Overlay Multicast

**DOI:** 10.1371/journal.pone.0142888

**Published:** 2015-11-12

**Authors:** Weidong Gu, Xinchang Zhang, Bin Gong, Wei Zhang, Lu Wang

**Affiliations:** 1 Department of Computer Science and Technology, Shandong University, Jinan, China; 2 Shandong Provincial Key Laboratory of Computer Networks, Shandong Computer Science Center(National Supercomputer Center in Jinan), Jinan, China; Nankai University, CHINA

## Abstract

Tree-based overlay multicast is an effective group communication method for media streaming applications. However, a group member’s departure causes all of its descendants to be disconnected from the multicast tree for some time, which results in poor performance. The above problem is difficult to be addressed because overlay multicast tree is intrinsically instable. In this paper, we proposed a novel stability enhancing solution, VMCast, for tree-based overlay multicast. This solution uses two types of on-demand cloud virtual machines (VMs), i.e., multicast VMs (MVMs) and compensation VMs (CVMs). MVMs are used to disseminate the multicast data, whereas CVMs are used to offer streaming compensation. The used VMs in the same cloud datacenter constitute a VM cluster. Each VM cluster is responsible for a service domain (VMSD), and each group member belongs to a specific VMSD. The data source delivers the multicast data to MVMs through a reliable path, and MVMs further disseminate the data to group members along domain overlay multicast trees. The above approach structurally improves the stability of the overlay multicast tree. We further utilized CVM-based streaming compensation to enhance the stability of the data distribution in the VMSDs. VMCast can be used as an extension to existing tree-based overlay multicast solutions, to provide better services for media streaming applications. We applied VMCast to two application instances (i.e., HMTP and HCcast). The results show that it can obviously enhance the stability of the data distribution.

## Introduction

Multicast is an ideal group communication method for media streaming applications. Existing multicast solutions can be divided into two main categories, i.e., IP multicast and overlay multicast (also called application layer multicast). Overlay multicast implements multicast functionality at the application layer, which overcomes some drawbacks (e.g., heavily depending on the support of multicast routers [1]) of IP multicast. Overlay multicast has been widely researched, and can be classified into mesh-based or tree-based solutions. Tree-based overlay multicast solutions [2–5] usually organize participating peers (group members) into one or more multicast trees, and disseminate the multicast data along these trees. Mesh-based overlay multicast solutions [6, 7] usually present multicast data as a series of segments, and each group member obtains the segments from some neighbors.

Mesh-based systems have lower data forwarding effectiveness compared to tree-based systems because they require periodical information exchange [8]. Bonald *et al*. [9] studied several tree-based overlay multicast solutions and showed that some can achieve near-optimal rate and delay in static streaming systems. However, tree-based overlay multicast systems are vulnerable to distribution instability, where a group member’s departure causes all of its descendants to be disconnected from the multicast tree for some time, which results in poor performance [10]. Since any group member can randomly leave a multicast session, distribution instability can be a significant issue, and is a challenge to address within the bandwidth and timing constraints for media streaming applications [11, 12]. A popular scheme to enhance the stability of tree-based overlay multicast is to build a stable tree structure. Solutions using this scheme can be classified into three main types—minimum-depth, proxy-based, and lifespan-based. In minimum-depth solutions (e.g. NICE [4] and ZigZag [13]), the new joining peer selects the neighbor of the minimum depth as its parent node. Proxy-based solutions (e.g. OMNI [14] and TOMA [15]) use additional proxy servers to assist in distributing the multicast data. These proxy servers are steady and can improve the multicast stability. The dynamics of peers in P2P systems has received wide attention [10, 16–18]. Lifespan-based solutions (e.g. LDTC [16], MDA [19] and ROST [20]) use peer dynamics feature to improve distribution instability. To summarize, the above solutions attempt to build relatively stable forwarding tree structure, but cannot make a control on the instability.

We proposed a VM-assisted stability enhancing solution (VMCast) for tree-based overlay multicast. In this solution, the used VMs are divided into two types, i.e., MVMs and CVMs. MVMs are used to disseminate the multicast data, whereas CVMs are used to offer streaming compensation. The used VMs in the same cloud datacenter constitute a VM cluster. Each VM cluster is responsible for a service domain, called VMSD, and each group member belongs to a specific VMSD. In VMCast, the data source delivers the multicast data to VMs through a reliable path, and MVMs further disseminate the data to group members in the corresponding VMSDs along an overlay multicast tree. Hence, the distribution instability problem of the whole group is transformed into distribution instability of sub-groups, which improves the stability of the overlay multicast tree. VMCast further employs a CVM-assisted streaming compensation method to overcome data interruptions mainly caused by departure of group members. VMCast can be used as an extension to existing tree-based overlay multicast solutions, to provide better services for media streaming applications. The two application instances (i.e., HMTP [2] and HCcast [3]) show that VMCast can obviously enhance the stability of the data distribution.

## Methods

### VMCast Structure

**Fig 1:**
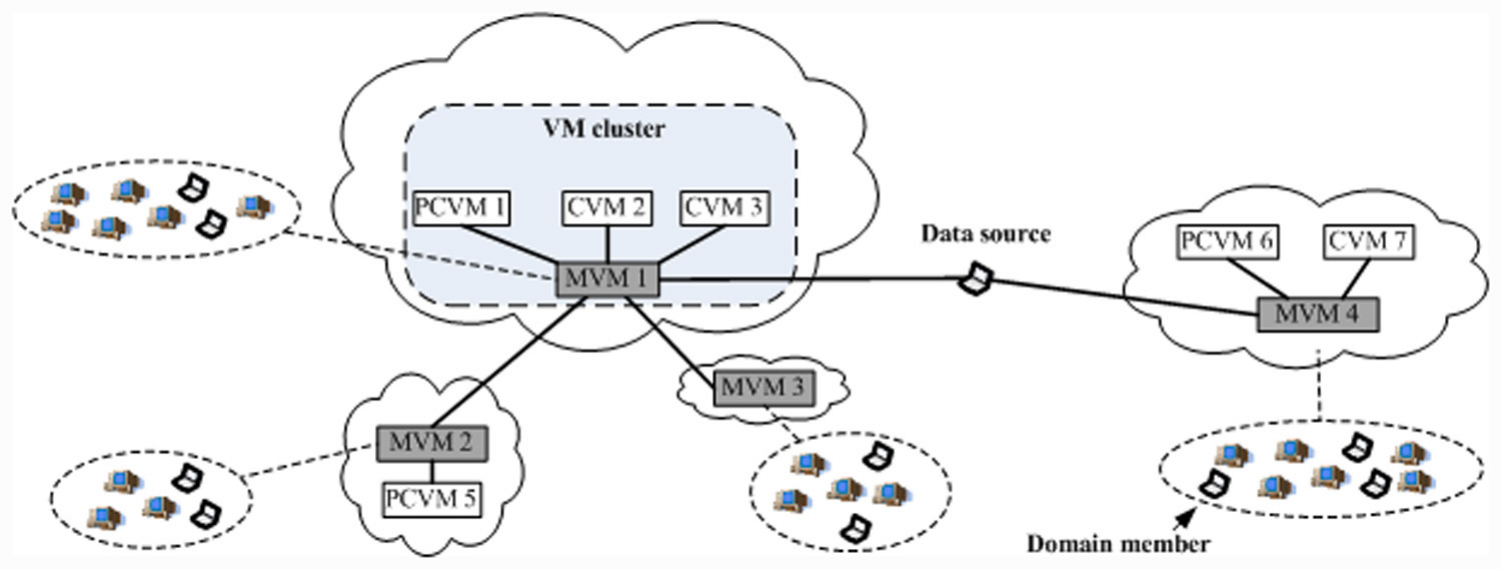
VMCast structure.

VMCast employs on-demand VMs in different cloud datacenters to enhance the stability of tree-based overlay multicast, as shown in Fig 1. The used VMs are divided into two types, i.e., MVMs and CVMs. As mentioned above, MVMs are used to disseminate the multicast data, whereas CVMs are used to offer streaming compensation. A cloud datacenter contains one MVM and *n* (*n* ≥ 0) CVMs. In a designated cloud datacenter, the first created VM is the MVM, and the first created CVM is called proxy CVM (PCVM). All the used VMs in the same cloud datacenter constitute a VM cluster. The MVM saves the address of the PCVM in the VM cluster which this MVM belongs to. Each VM cluster is responsible for a VMSD, and each group member belongs to the VMSD which the closest VM cluster is responsible for. A VM cluster is said to be the associated VM cluster of each group member in the VMSD which this cluster is responsible for.

In VMCast, the multicast data is distributed in a two-tier manner. In the first tier, the multicast data is delivered to MVMs along an overlay multicast tree, and each MVM forwards the received data to CVMs in the same VM cluster one by one. This structure is called the core overlay tree. The data distribution along the core overlay tree is reliable because there are no dynamic group members in the tree. The data source can distribute the multicast data to used VMs in advance or on demand. In the second tier, each MVM provides a best-effort data distribution service, disseminating the received multicast data to group members in the corresponding domain one by one if it has enough bandwidth. If an MVM has insufficient bandwidth, then it disseminates the multicast data along an overlay multicast tree (called domain overlay tree). A domain overlay tree is rooted by an MVM (denoted by *v*) and includes the group members in the VMSD which *v*-included VM cluster is responsible for. Thus, the distribution instability of the whole group is transformed into distribution instability of the sub-groups in the VMSDs, which can improve the stability of tree-based overlay multicast.

Let *dn*(*p*) denote the number of downstream nodes of a tree node *p*. We can deduce the following theorem.

**Theorem 1** *Given an overlay multicast tree with m nodes. Assume that 1) M means the set of all the nodes except the root, and 2) k denotes the maximum out-degree (i.e., fanout) of all the nodes, then* $\sum_{p\in M}dn\left(p\right)\geq \left(u-1\right)m-\frac{u-1}{1-k}+\frac{k-k^{u}}{{\left(1-k\right)}^{2}}$, *where u* = ⌈*log*_*k*_ *m*⌉.

**Proof 1** *Assume that there are u levels in the overlay multicast tree (denoted by T). We use ln*(*L_i,T_*) *to denote the number of the nodes at level i* + 1, *i* + 2, ⋯, *u in the tree. Note that the tree root is at level 1. We can have*

$$\sum_{p\in M}dn\left(p\right)=\sum_{i=1}^{u}ln\left(L_{i,T}\right).$$ (1)

*Comparing the complete* *k-ary tree* $T_{k}^{{}^{\circ}}$ *(in which every internal vertex has exactly k children) with any a* *k-ary tree* *T*_*k*_, *we can notice that 1) the the highest level of* $T_{k}^{{}^{\circ}}$ *are not more than that of* *T*_*k*_, *and 2)* ∀*i*(1 ≤ *i* ≤ ⌈*log*_*k*_ *m*⌉), $ln\left(L_{i,T_{k}^{{}^{\circ}}}\right)\leq ln\left(L_{i,T_{k}^{{}^{\prime }}}\right)$. *Thus we can have* $\sum_{p\in M}dn\left(p\right)\geq \sum_{i=1}^{u}ln\left(L_{i,T_{k}^{{}^{\circ}}}\right)$. Let *u* = ⌈*log*_*k*_ *m*⌉, *we can deduce that*

$$\begin{array}{c} \sum_{i=1}^{u}ln\left(L_{i,T_{k}^{{}^{\circ}}}\right)=\left(u-1\right)m-\frac{u-1}{1-k}+\frac{k-k^{u}}{{\left(1-k\right)}^{2}} \end{array}$$ (2)

*This theorem has been proven*.

As mentioned previously, a group member’s departure will cause all of its descendants to become disconnected from the overlay multicast tree for some time, which results in some data losses. We use $\sum_{p\in M}dn\left(p\right)$ to evaluate the negative influence of group members’ departures. From Theorem 1, we can see that the negative influence of group members’ departures weakens as the group size decreases. VMCast can effectively alleviate the negative influence of group members’ departures because it uses reliable core overlay tree to divide the whole group into some small-sized sub-groups.

Unlike physical servers, cloud VMs can be created on demand in terms of practical requirement. The cloud providers usually charge for VMs by the using time. For example, an EC2 VM (RAM: 3.75, vCPUs: 1, Disk: 410GB) is charged by $0.12/hr. Thus the use cost of VMs is low. In general, it requires only a few minutes to create a new VM. Therefore the streaming compensation capability can be easy extended by adding new CVMs. Network traffic consumed by a VM is usually calculated separately. In most clouds, there is no charge for incoming traffic and traffic within private networks. Generally, the cloud provider aggregates outgoing network traffic by cloud accounts, and invoices users on a monthly basis. Paying only for used traffic is very advantageous for VMCast service because the streaming compensation requirement is difficult to be predicted.

### VM-assisted Overlay Multicast Tree

Some VMs should be deployed in advance, depending on the group scale and group member location distribution of served multicast application. Deploying a few more VMs in the initial stage is feasible because the machine cost of a VM is low. If the approximate locations of group members are known, then VMs are created from cloud datacenters close to group members; otherwise, VMs are created from the cloud datacenters in scattered locations. Note that each VM cluster includes at least one MVM, and usually includes multiple CVMs.

The VMCast multicast tree includes an overlay multicast tree which connects different MVMs, star-like trees which each connect the MVM and CVMs in the same VM cluster, and domain overlay trees. The overlay multicast tree connects different MVMs is built by existing tree-based overlay protocol. Each MVM forwards multicast data to CVMs in the same VM cluster one by one, which forms a star-like tree.

In this paper, the maximum outdegree of a VM *v* denotes the maximum number of the peers which simultaneously obtain streaming from *v*. Note that *v*’s maximum outdegree depends on *v*’s total bandwidth and the required bandwidth of the multicast session. Let *a* and *b* be any two VMs in the same VM cluster and *m* means a peer, we reasonably assume that *d*(*a*, *m*) = *d*(*b*, *m*). Let *M* denote the set of all the MVMs. A PCVM *v* is said to be a candidate compensation PCVM of *m* if *d*(*v*, *m*), the distance between *v* and *m*, satisfies

d(v,m)≤min{d(b,m)|b∈M}+Δ, (3)

where Δ is a distance deviation bound. Note that *m*’s potential candidate compensation CVMs increase with the growth of Δ, and vice versa.

When a peer *m* wants to join a multicast session, it measures the distances between MVMs and itself, and requests for the PCVMs of VM clusters from MVMs. Then *m* selects the closest candidate MVM as its associated MVM (denoted by *av*(*m*)), and inserted candidate compensation PCVMs into a link (called enhancing link) as the ascending order of distance. Next *m* sends a *Join* message to *av*(*m*). If *av*(*m*) has remaining outdegree, then it accepts *m* as its child node; otherwise, *m* connects to the domain overlay tree rooted by *av*(*m*) using an existing overlay multicast protocol.

**Fig 2:**
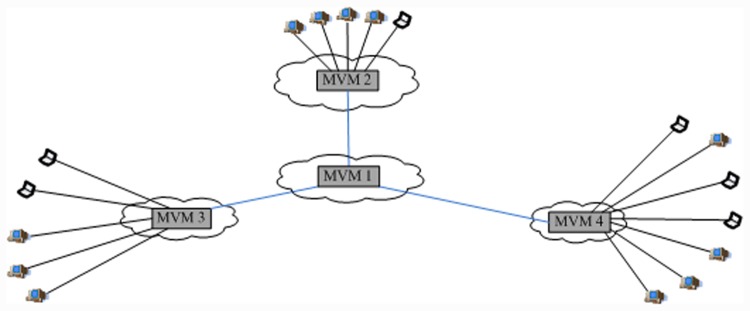
Tree-star structure of VMCast.

If the total bandwidth of MVMs is enough to accommodate all the group members, then the VMCast multicast tree becomes a tree-star structure, as shown in Fig 2. In this structure, stability is very high because the dynamics of any group member has no influence on other members. The tree-star multicast structure is applicable to some jitter-sensitive and interactive streaming applications (e.g. video conference) with a small amount of users. However, the cost of tree-star multicast structure is high in the case where the group includes a large number of members. VMCast uses a streaming compensation mechanism to obtain high stability with relatively low cost (see the following section).

## Streaming Compensation

In the group application, group members can randomly leave the multicast session. When a group member leaves the multicast session, the data distribution to all its descendants is interrupted until the new multicast tree is built, which make the data distribution instability. The VM-assisted structure improves the stability of whole overlay multicast tree through reducing the influence scope of a data distribution interruption. However, it cannot fundamentally solve the data distribution instability problem. We introduce streaming compensation method to address the above problem.

**Fig 3:**
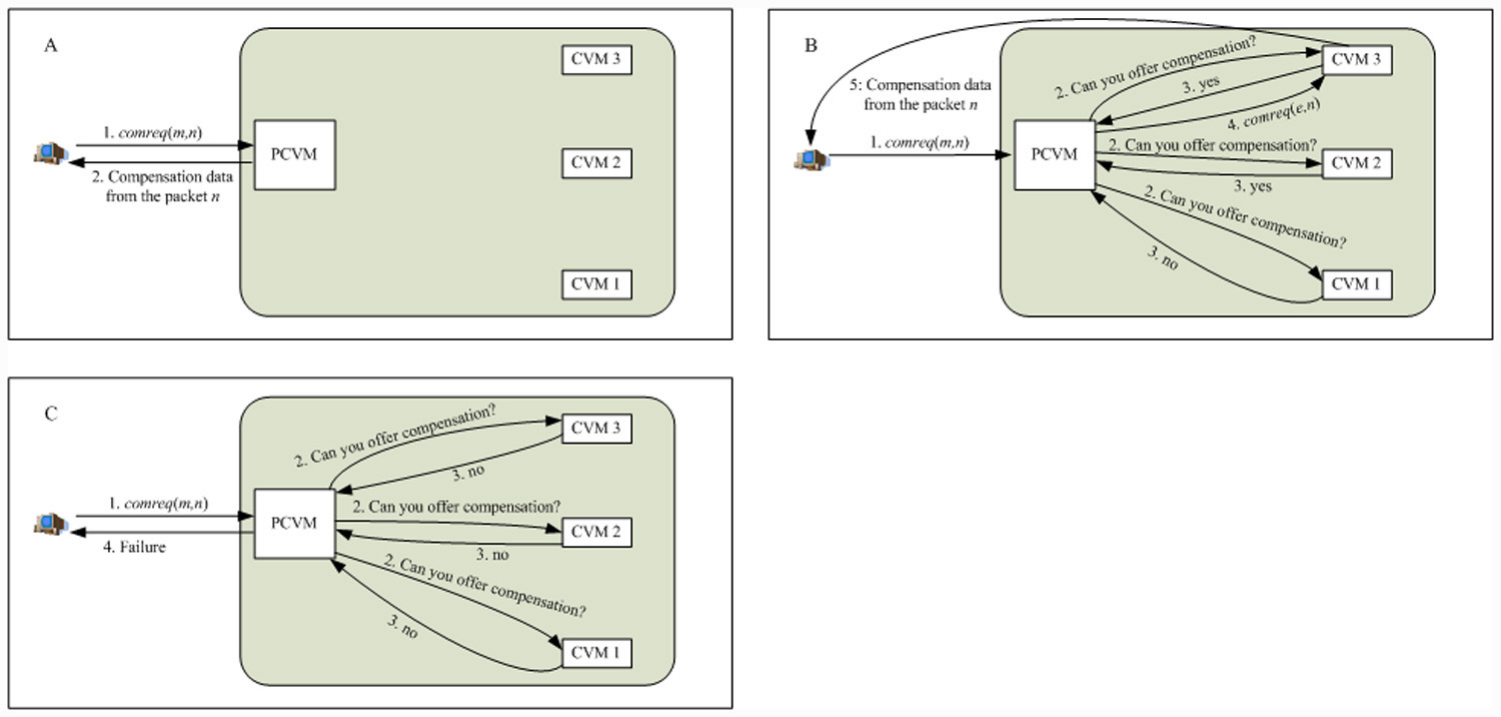
CVM-based streaming compensation.

In VMCast, each group member *m* real-timely monitors received multicast flow. If a group member finds that it have not received the next expected packet for a designated time (e.g., 100ms), then it determinate that the streaming interruption occurs. When *m* detects the data interruption, then it sends a streaming compensation request *comreq*(*m*, *n*) to the first CVM *v* of *m*’s enhancing link, where *n* denotes the sequence number of interrupted packet. If *v* has remaining outdegree, then it directly offers streaming compensation to *m*, as Fig 3A shows. If *v* has no remaining outdegree but some CVMs in the same VM cluster have remaining outdegree, then a random CVM, which has remaining outdegree, is appointed to offer streaming compensation to *m*. Fig 3B explains the above procedure. If all the CVMs (including the PCVM) in the VM cluster, which *v* belongs to, has no remaining outdegree, then *m* chooses the next PCVM in its enhancing link and sends *comreq*(*m*, *n*) to it. The above procedure goes on until (1) *m* obtains the corresponding streaming compensation, or (2) no appropriate CVM can offer the streaming compensation, or (3) *m* does not further send the streaming compensation request because it can normally receive the multicast data from the overlay multicast tree again. A group member tells the CVM, which is providing streaming compensation service for it, to stop streaming compensation when it can normally receive the multicast data from the domain overlay tree again.

From the above description, we can notice that the VMs in a designated cloud datacenter cooperatively offer streaming compensation for group members. Note that these VMs can communication with each other with very low latency because they are in the same private network. Consider another possible data compensation scheme, called independent data compensation, in which data compensation requests are randomly sent to VMs. In the independent data compensation scheme, the VM directly refuses new data compensation requests if it has no enough remaining bandwidth. We can have the following theorem.

**Theorem 2** *Assume that (1) a cloud datacenter includes* *m* *VMs, each of which can offer data compensation for at most* *σ* *group members, and (2)* *n* *data compensation requests are randomly sent to the* *m* *VMs, then the probability that at least one data compensation request is refused, denoted by* *F*(*n*, *m*, *σ*), *is* $\frac{P_{m}\left(n-\sigma -1+m\right)}{P_{m}\left(n+m\right)}$. *Note that* *P*_*m*_(*n* + *m*) *denotes the number of* (*n* + *m*)’s *m-partitions which each include* *m* *components*.

**Proof 2** *Randomly sending* *n* *data compensation requests to at most* *m* *VMs can be thought as of placing* *n* *identical balls into at most* *m* *identical boxes. The number of the above ball placement schemes is* *P*_*m*_(*n* + *m*). *Randomly sending* *n* *data compensation requests to at most* *m* *VMs, such that at least one data compensation request is refused, can be thought as of placing* *n* *identical balls into at most* *m* *identical boxes such that at least one box contains at least* *σ* + 1 *balls. The latter can be done as the following two steps:* *σ* + 1 *identical balls are placed into some box;* *n* − *σ* − 1 *identical balls are placed into at most* *m* *identical boxes. Thus the number of placement schemes, that place* *n* *identical balls into at most* *m* *identical boxes such that at least one box contains at least* *σ* + 1 *balls, is* *P*_*m*_(*n* − *σ* − 1 + *m*). *Therefore* $F\left(n,m,\sigma \right)=\frac{P_{m}\left(n-\sigma -1+m\right)}{P_{m}\left(n+m\right)}$. *This theorem has been proven*.

From Theorem 2, we can notice that *P*_*m*_(*n* + *m*)>0 if *n* > *σ*. Compared with the independent data compensation scheme, our scheme can respond to each of the *n* data compensation requests with corresponding data compensation in the case where *n* ≤ *mσ*.

## Results

### Simulation Experiments

We used the GT-ITM Generator [21] to create a 5,000-node transit-stub graph as our underlying network topology, and conducted simulation experiments using the NS2 [22] simulator. The VM clusters and peer nodes were randomly connected to stub-domain nodes. The maximum outdegree of each peer was set to a random integer from 2 to 4, and the maximum outdegree of each VM was set to a random integer from 10 to 20. We investigated VMCast systems using HMTP [2] and HCcast [3] to build the overlay tree connecting MVMs and domain overlay trees, respectively. We use VMCast-*X* to denote the VMCast system using the overlay multicast solution *X* to build the core overlay tree connecting MVMs and domain overlay trees. In our experiments, each related cloud datacenter built 1 MVMs and 2 CVMs for a designated multicast session.

The transfer time ratio was used to evaluate the performance of VMCast. Transfer time ratio compares an overlay multicast solution, *A*, and VMCast-*A* in the duration of the data interruption caused by the group members dynamics. The transfer time ratio for a group member, *m*, in VMCast-*A* is

$$tr\left(m,A\right)=\frac{itt(m,A)}{itt(m,\text{VMCast-}A)},$$ (4)

where *itt*(*m*, *X*) is the duration from the moment that *m* detects the streaming interruption to the moment that *m* re-receives the data packets in the solution *X* (*A* or VMCast-*A*).

**Fig 4:**
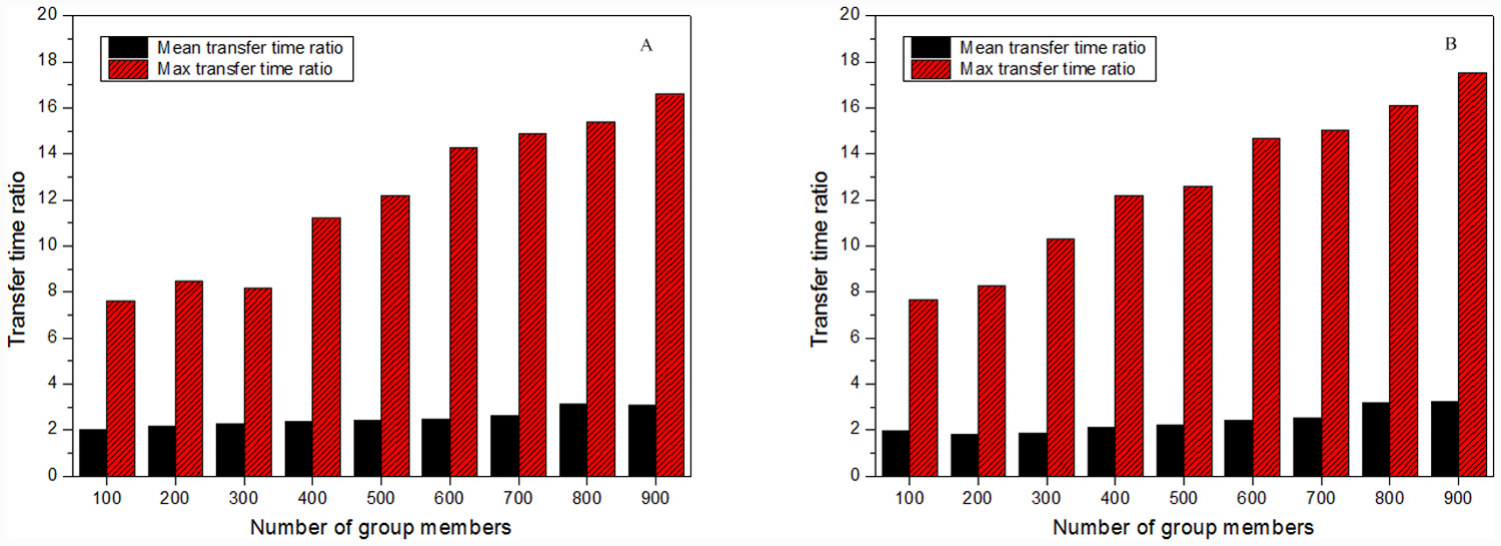
Transfer time ratios of 9 multicast sessions. (A) The results of VMCast-HMTP, and (B) The results of VMCast-HMTP.

**Fig 5:**
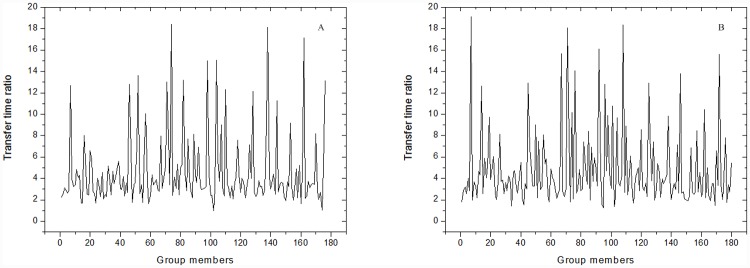
Transfer time ratios of the session with 900 group members. (A) The results of VMCast-HMTP, and (B) The results of VMCast-HMTP.

We investigated 9 different multicast sessions, which each used three VM clusters. The group sizes (i.e., the numbers of group members) ranged from 100–900, increasing by 100. In each multicast session, about 6% group members left the group at a random moment. Fig 4 shows the maximum and mean transfer time ratios in the 9 sessions. The mean transfer time ratios in VMCast-HMTP and VMCast-HCcast both exceed 2.0, which indicates that VMCast alleviates distribution instability caused by group members’ dynamics. With the growth of the group size, transfer time ratios in VMCast-HMTP and VMCast-HCcast tend to increase. This is mainly due to group members taking longer to reconnect to the multicast tree as the group size increases. Fig 5 shows the transfer time ratios in VMCast-HMTP and VMCast-HCcast in the multicast session with 900 group members. VMCast significantly improves the data distribution stability.

**Fig 6:**
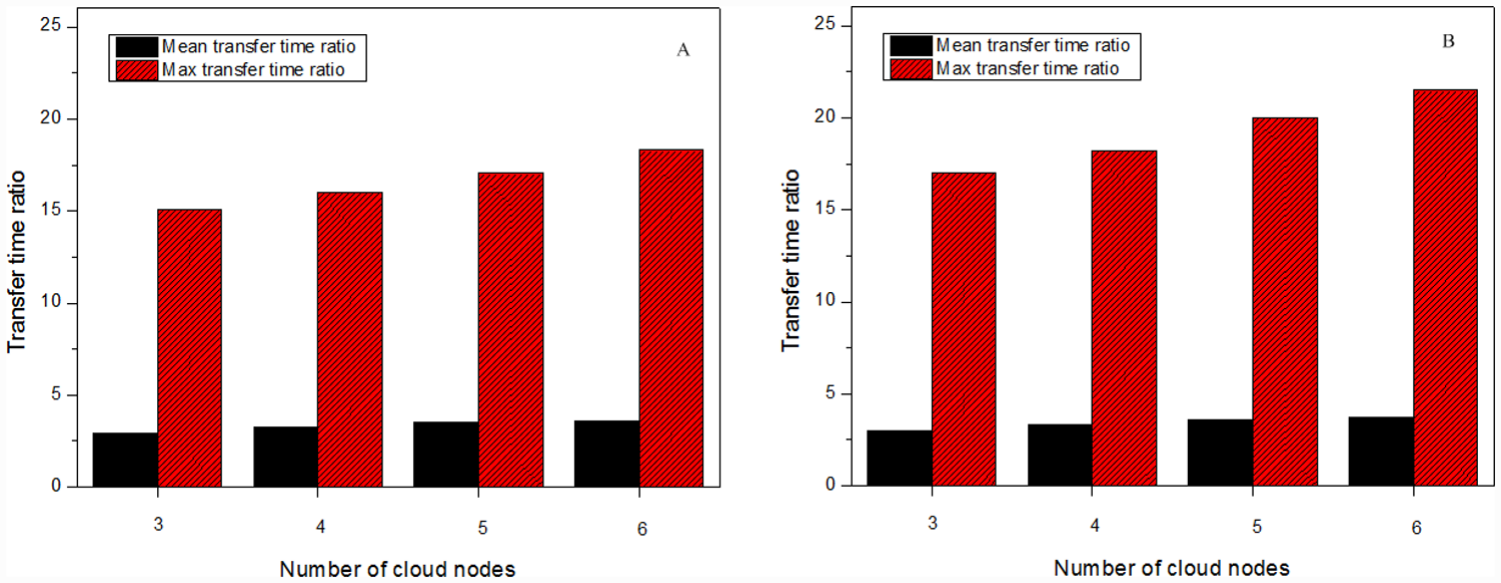
Transfer time ratios with different VM clusters. (A) The results of VMCast-HMTP, and (B) The results of VMCast-HMTP.

**Fig 7:**
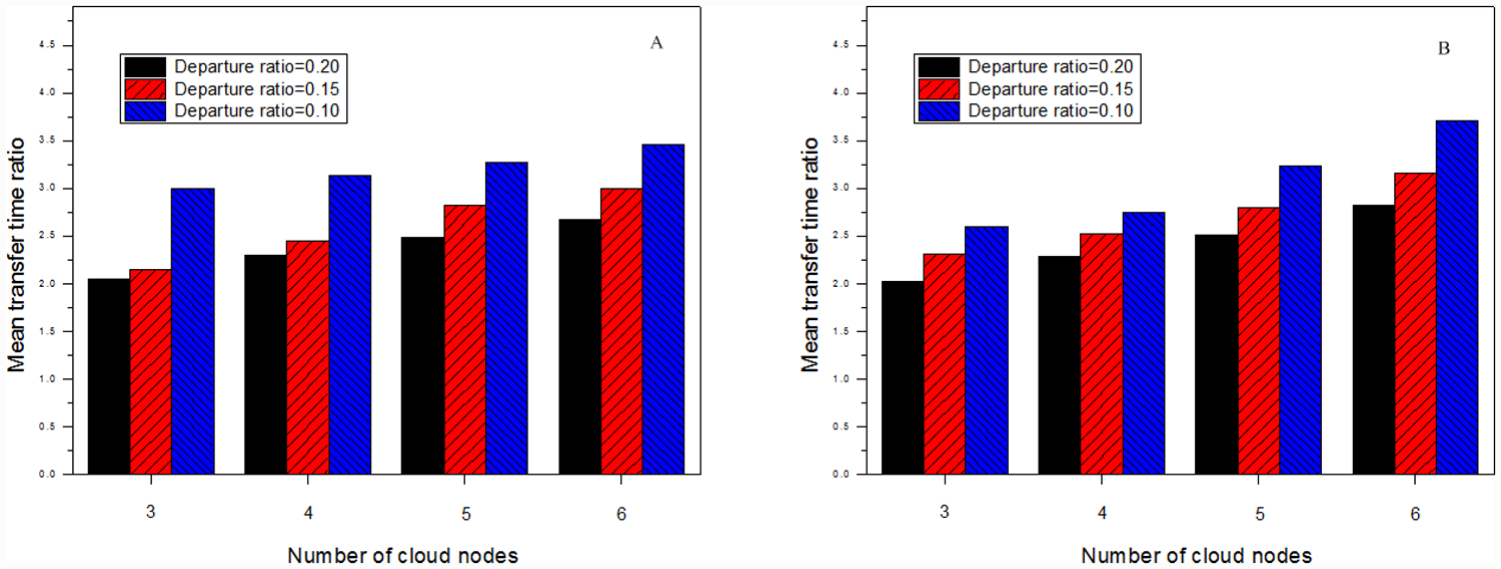
Transfer time ratios with different departure ratios. (A) The results of VMCast-HMTP, and (B) The results of VMCast-HMTP.

Fig 6 shows transfer time ratios in the multicast sessions supported by different numbers of VM clusters. Each session includes 900 group members. As the number of VM clusters grows, transfer time ratios in VMCast-HMTP and VMCast-HCcast tend to increase. Fig 7 further describes transfer time ratios of multicast groups with different departure ratios, which denotes ratio of the number of group members which leave the multicast session to that of all group members. We can notice that more VM clusters can better improve the data distribution instability caused by the group member dynamics.

**Fig 8:**
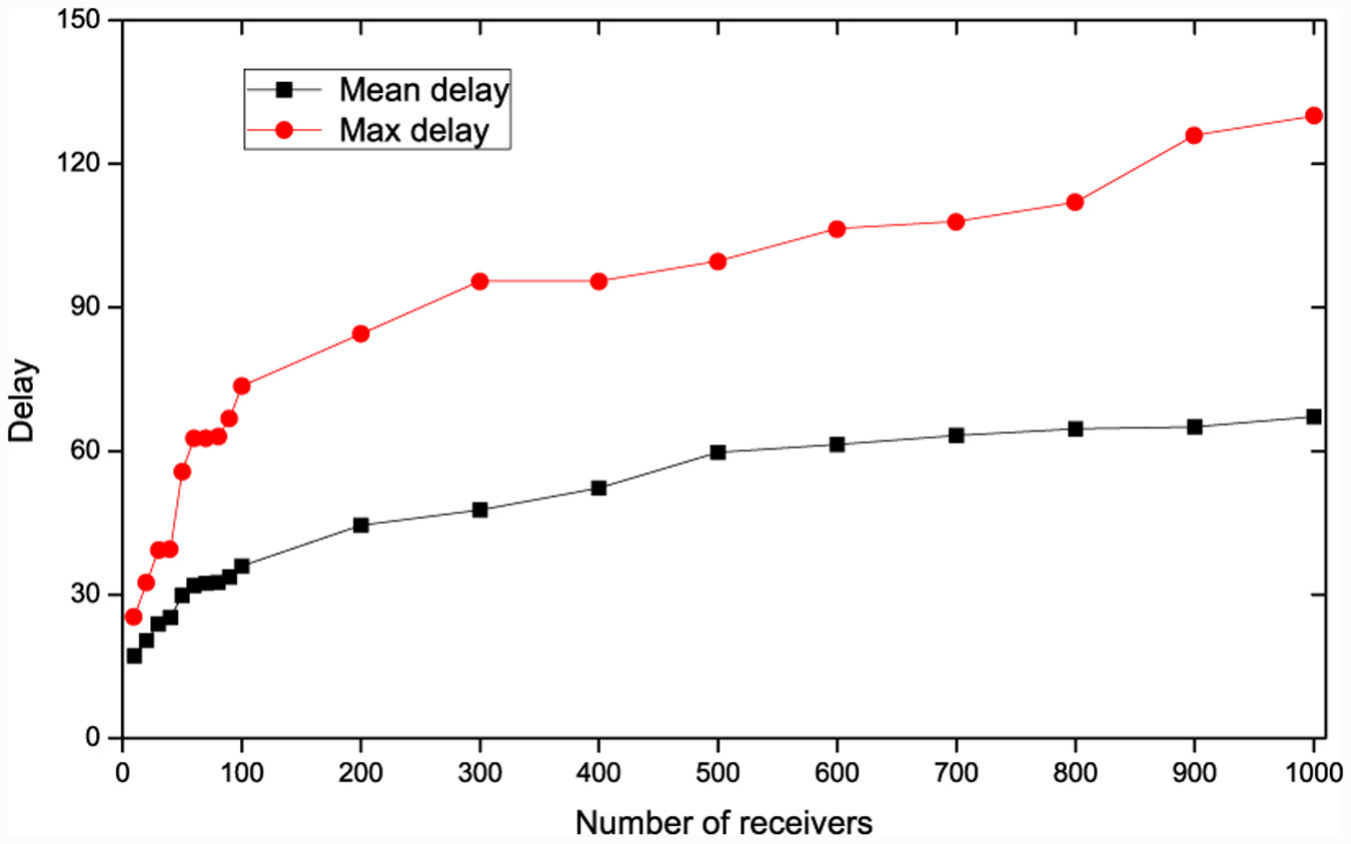
Data distribution delays of HCcast trees with different group members.

Where the multicast data is delivered to the MVMs in advance, the data distribution delay completely depends on domain overlay trees. Generally, the data distribution delay is lower in the smaller group, as shown in Fig 8. Therefore, VMCast also reduces data distribution delay in this case.

### Internet Experiments

**Fig 9:**
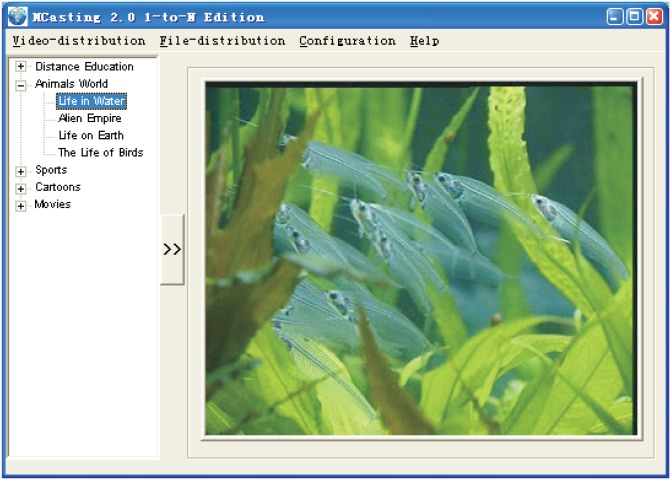
Overlay multicast experimental system.

We conducted some real experiments to investigate the performance of VM-assisted data distribution, using our developed overlay multicast experimental system shown in Fig 9. We obtained 11 VMs from the clouds: EC2 (US), Rankspace (US), Aliyun (China), 400apps (China), and Ksyun (China). Each VM service domain included 10 to 20 group members.

Table 1 lists the packets’ arrival time intervals at 50 group members. Our data show that the VM-assisted data distribution has high stability in the real, internet based situation.

**Table 1:** Arrival time intervals (ms) of date packets to group members.

**Associated cloud**		**Group member number**									
		1	2	3	4	5	6	7	8	9	10
EC2	Max	75	79	79	107	108	109	112	109	99	79
	Avg	55	56	58	57	58	58	60	71	66	55
	Min	35	34	34	34	31	31	31	35	32	31
Rackspace	Max	118	83	93	101	103	112	113	113	113	83
	Avg	57	58	58	59	58	63	64	62	64	58
	Min	31	32	32	32	32	31	32	31	32	32
Aliyun	Max	113	108	117	98	101	104	98	101	101	107
	Avg	62	62	65	65	66	65	55	61	60	64
	Min	32	32	32	31	31	31	31	31	31	31
400apps	Max	108	118	108	78	106	104	114	108	104	106
	Avg	63	68	63	56	54	62	63	62	61	61
	Min	31	31	31	34	31	31	31	31	31	31
Ksyun	Max	104	109	107	106	108	118	115	115	117	117
	Avg	61	62	61	62	71	70	71	69	70	71
	Min	31	31	31	31	32	32	32	32	31	31

## Conclusion

VMCast can be used as an extension to existing tree-based overlay multicast solutions, to provide better stable services for media streaming applications. In VMCast, the data source disseminates the multicast data to used VMs along a stable overlay multicast tree, and some VMs further disseminate the data to group members along overlay multicast trees. This above method make the overlay multicast tree more stability. VMCast further enhances the stability of the data distribution through dynamic streaming compensation based on some special VMs. Unlike existing approach, VMCast address the instability of tree-based overlay multicast in both active and passive manners.

## Supporting Information

S1 File Raw data of the data packet arrival times in the Internet experiments. (XLSX)
